# Master’s level mental health nursing competencies, a prerequisite for equal health among service users in mental health care

**DOI:** 10.1080/17482631.2018.1502013

**Published:** 2018-08-01

**Authors:** Henrika Jormfeldt, Louise Doyle, Heikki Ellilä, Mari Lahti, Agnes Higgins, Brian Keogh, Oonagh Meade, Theodore Stickley, Jan Sitvast, Ingela Skärsäter, Nina Kilkku

**Affiliations:** a School of Health and Wellfare, Halmstad University, Halmstad, Sweden; b School of Nursing and Midwifery, Trinity College Dublin, Dublin, Ireland; c Health and Well-being, Turku University of Applied Science, Turku, Finland; d School of Health Sciences, Faculty of Medicine & Health Sciences, University of Nottingham, Nottingham, UK; e Advanced Nursing Practice, University of Applied Sciences HU, Utrecht, The Netherlands; f Tampere University of Applied Sciences, Tampere, Finland

**Keywords:** Holistic health, “master’s level mental health nursing competencies”, mental health care, physical health

## Abstract

**Purpose:** This discussion paper aims to explore the need of a clarified definition of master’s level mental health nursing competencies in terms of knowledge, skills and attitudes in a European context. Mental health service users have, in spite of their right to equal overall health, higher rates of physical illness and are more likely to experience premature death than the general population. Implementation of a holistic concept of health comprising mental, physical and social aspects of health in mental health services has previously proved to be challenging. **Methods:** Master’s level mental health nursing competencies in recent literature are discussed and illuminated in terms of knowledge, skills and attitudes in order to enable the promotion of equal overall health among service users in mental health services. **Results:** The discussion show contents, values and utility of master’s level mental health nursing competencies in mental health services and contribute to reduced role ambiguity by distinguishing master’s level responsibilities from undergraduate nursing tasks and obligations of other professionals in mental health care. **Conclusion:** This discussion paper shapes implications for developments in master’s level mental health nursing education curricula.

## Introduction

Individuals experiencing enduring mental health issues have higher rates of physical illness and are more likely to experience premature death than the general population (Blyte & White, 2012). General health-promotion praxis available for people experiencing long lasting mental health issues has been described as insufficient (Lambert & Newcomer, 2009), and experiences of health-promotion interventions described by persons experiencing severe mental health issues are rare (Ehrlich, Kendall, & Frey et al., 2014). In addition, physical ill health among mental health service users has repeatedly been mistaken for symptoms of “mental illness” and overlooked, even by mental health nurses (Bradshaw & Pedley, 2012). A clarification of responsibilities among different professions, and the specific obligations of mental health nurses in relation to services users’ needs of health promotion, may be a prerequisite to overcome the shortcomings of overall and physical health promoting activities in mental health services (Gunnmo & Fatouros Bergman, 2011; Hodgson, McCulloch, & Fox, 2011). The context for this article is a European-funded international project, developing master’s level eLearning materials for education regarding mental health (eMenthe). As a part of the project, this specific paper focuses on master’s level competencies of mental health nurses’ regarding the physical health needs of people who use mental health services.

## Background

The traditional mental healthcare system in many countries has a normative orientation, with a focus on the reduction of psychiatric symptoms and the prevention of relapses (Svedberg, Jormfeldt, & Fridlund et al., 2004; Van Wel & Landsheer, 2011). Conceptual differences regarding the meaning of mental health and recovery between European countries have been found, but a common perspective among most counties is to adopt a traditional view of these concepts as a reduction of symptoms rather than being about regaining the ability to live a good life regardless of symptoms (Keogh, Doyle, & Ellilä et al., 2017). A strict provider-centred approach is predominantly focused on medical treatment of mental illness, which may not automatically increase health, well-being and an ability to achieve personal goals (Chee, 2009). The experience of being a service user in a psychiatric context has been described by former service users as being constrained within a structure of control by a “common staff approach” characterized by power and authority (Enarsson, Sandman, & Hellzeén, 2011).

Accordingly, health education for people using mental health services generally have a provider-centred focus mainly aiming to increase patients’ knowledge about their mental illness and treatment (Brouse, Basch, & Kubara, 2005; Pekkala & Merinder, 2009). A review of international schizophrenia research conferences performed by Calton, Cheetham, and D’Silva et al. (2009) between 1988 and 2004 revealed that biological research was the main theme of 6,960 (75%) of the abstracts, while psychosocial research constituted less than 5% and only 2% were service user-centred. This situation concurs with the basic concepts in the traditional disease oriented model and places mental health nurses in difficult situations having to provide nursing interventions in a context where the “provider–patient” relationship historically has been controlling or paternalistic in nature (Anderson & Funnell, 2005; Brouse et al., 2005).

The World Health Organization (1991) has stated that the concepts of health and health potential include both physical and mental health and must be seen in the context of personal development through life. Health has shown to be positively related to subjectively experienced self-esteem, empowerment and quality of life, and only to a minor extent adversely related to psychiatric symptoms (Jormfeldt, Arvidsson, & Svensson et al., 2008). Mental health nursing competencies are built on the fundamental understanding of a holistic concept of health to address clinical interventions that aim at strengthening positive dimensions of health in order to meet the service users’ needs of enhanced overall health (Jormfeldt, 2011; Verhaeghe et al., 2013a; Verhaeghe, De Maeseneer, Maes, Van Heeringen, & Annemans, 2011). Overall health promotion, including facilitating mental, physical, and social health among service users remains an important component of mental health nursing practice at both individual and societal levels (Doyle, Ellila, & Jormfeldt et al., 2017).

The Quality and Safety Education for Nurses (QSEN) project at Cape Western Reserve University in USA has been working to implement six core competencies in teaching as well as in daily care (Cronenwett, Sherwood, & Pohl et al., 2009). The six core competencies are described as: Quality Improvement, Safety, Teamwork and Collaboration, Patient-centred Care, Evidence-Based Practice and Informatics. The overall goal of the work by QSEN has been to meet the challenge of preparing future nurses worldwide to have the knowledge, skills and attitudes (KSAs) necessary to continuously improve the quality and safety of the healthcare systems within which they work (Cronenwett et al., 2009). However, the predominant biomedical paradigm of mental health education and practice has predestined that many nurses have not been sufficiently equipped to integrate a holistic perspective of health with a focus on wellness and resilience, rather than deﬁcits, into their mental health practice. Findings, from interviews with stakeholders across ﬁve European countries, suggest a need to reorientate mental health nursing education to equip mental health nurses with the skills needed to work with regard to the services users’ needs (Doyle et al., 2017). In this discussion paper, project partners from five European counties were inspired by the QSEN’s KSAs to distinguish the knowledge, skills, and attitudes required by master-level mental health nurses to practice within a paradigm of holistic health with respect to mental health service users’ right to equal physical health. The purpose of this paper was thus to discuss master’s level mental health nursing competencies in terms of knowledge, skills and attitudes required to meet mental health service users’ right to physical health in a European context.

## Master’s level mental health nursing competencies in terms of knowledge, skills and attitudes knowledge

Three essential domains of knowledge needed by master’s level mental health nurses to facilitate and support equal health among service users in mental health care could be described as: specific knowledge about physical health risks connected with mental illness; detailed knowledge about mental illness stigma and its effects on physical health; and extensive knowledge of mental health nurses’ duty to promote mental and physical health in mental health service users.

### Specific knowledge about physical health risks connected with mental illness

The prevalence of poor physical health and health behaviours in people with enduring mental health difficulties far exceed that which is observed in the general population owing to risk factors as cigarette smoking, obesity leading to dyslipidaemia, insulin resistance as well as diabetes, and hypertension (De Hert, Schreurs, & Vancampfort et al., 2009; Gothefors, Adolfsson, & Attvall et al., 2010; Simonelli-Muñoz, Fortea, & Salorio et al., 2012). Schizophrenia is associated with premature mortality with unclear causes mainly because of avoidable lifestyle related to ischaemic heart disease and cancer. The average life expectancy of the general population is 76 years, whereas the corresponding figure is 61 years among people with a diagnosis of schizophrenia (Hennekens, Hennekens, & Hollar et al., 2005). More than two-thirds of individuals diagnosed with schizophrenia, compared with approximately one-half in the general population, die of coronary heart disease (Crump, Winkleby, & Sundquist et al., 2013). People with a diagnosis of schizophrenia often, for a variety of reasons, have unhealthy dietary habits, as indicated by the findings of the study of Simonelli-Muñoz et al. (2012), which show that 51% of the participants took no longer than 15 min to eat, 40.8% did not eat fruit daily, and 63.1% did not eat fish. These dietary patterns are often combined with a low frequency of physical activity low self-estimated general state of health (Lassenius, Åkerlind, & Wiklund-Gustin et al., 2013), associated with an increase in body mass index, waist circumference and development of obesity with related metabolic alterations (Simonelli-Muñoz et al., 2012). Poor dietary patterns may be due to a combination of financial shortcomings and lack of skills in food preparation in spite of motivation to improve health (Hardy & Gray, 2012). Even though studies are inconclusive, there is a lot of evidence that long-term exposure to antipsychotics increases mortality in schizophrenia (De Hert et al., 2009; Gothefors et al., 2010; Weinmann, Read, & Aderhold, 2009). Hence, there is an urgent need for physical health concerns to be addressed, and master’s level mental health nurses need sufficient knowledge to be able to take on a pronounced responsibility to prevent the metabolic side effects of medication and poor general health in mental health service users (Scott & Happell, 2011).

### Detailed knowledge about mental illness stigma and its effects on physical health

Schizophrenia is one of the most stigmatized psychiatric diagnoses, and the increased frequency of physical diseases among individuals diagnosed with schizophrenia might be related to the unsatisfactory organization of health services, attitudes of medical personnel and the social stigma attributed to people with such a diagnosis in society (Leucht, Burkard, & Henderson et al., 2007). Three different types of stigma have been identified as barriers to healthy living; structural stigma, social stigma and self-stigma (Graham, Griffiths, & Tillotson et al., 2013). Structural stigmas refer to how mental health services are organized often apart from general health care and decreasing availability to general health care among mental health service users. Social stigmas raise how mental health service users often are viewed in an undesirable way in society, and self-stigmas integrate all types of stigma leading to a negative self-image of incompetence among service users (Graham.et al., 2013). Significant barriers to physical activity among persons with mental health problems, beside symptoms of mental illness, medications and weight gain from medications, also include fear of discrimination and safety concerns (Nyeboe & Lund, 2013). Previous studies have confirmed that persons with enduring mental health issues often live a sedentary and inactive life, and mental health nurses need to emphasize the importance of physical activity and promote structured routines including physical activities as an essential part in recovery (Leutwyler, Hubbard, & Slater et al., 2014; Nyeboe & Lund, 2013; Ussher, Stanbury, & Cheeseman et al., 2007). Master’s level mental health nurses thus need to have profound knowledge of the ultimate consequences of taboos and prejudices on different levels in society, including inequality in access to general healthcare, to be able to possess a distinct and manifest liability to counteract prejudgements regarding mental health service users.

### Extensive knowledge of mental health nurses’ duty to promote mental and physical health in mental health service users

Insufficient priority has been given to meet the physical health-care needs of people experiencing mental illness (Heald, Montejo, & Millar et al., 2010; Laursen & Nodentoft, 2006) Mental health nurses, owing to their competence profile, have a key role in improving overall health and well-being of people with mental illness (Hardy & Thomas, 2012) comprising preventing, detecting, and managing side effects of antipsychotics (Usher, 2006). People with mental health issues, in spite of the will to learn more about healthy lifestyles, often lack the ability to change their physical health on their own, putting them in need of the mental health nurses’ emotional, practical and mutual support (Verhaeghe et al., 2011). Such supports are often most significant in order to handle lack of readiness among individuals with enduring mental ill health (Aschbrenner et al., 2103). Physical activity is known to improve overall health, including mental well-being, physical health and social involvement among mental health service users (Montejo, 2010). It is crucial that master’s level mental health nurses have the basic knowledge that physical activity is important to reduce anxiety and stress (Erdner & Magnusson, 2012). Health promotion for this population ought to capitalize on therapeutic alliances with staff and informal peer networks (Kemp, 2015). Master’s level mental health nurses have an important role in assisting the individual to engage in these supportive networks and the master’s level mental health nursing knowledge involves the essential understanding to be able to see the service user as a whole human being irrespective of psychiatric condition (Blomqvist, Sandgren, & Carlsson et al., 2018).

## Skills

Two main skills needed in master’s level mental health nursing to facilitate and support equal health among service users in mental health care are: advanced therapeutic and pedagogical skills to motivate healthy living; and teamwork and collaboration skills to counteract barriers to a healthy lifestyle among mental health service users.

### Advanced therapeutic and pedagogical skills to motivate healthy living

Healthcare professionals, in particular master’s level mental health nurses, need to consider matters that may hinder or encourage individuals with mental health issues to participate in lifestyle interventions to achieve sufficient benefits (Roberts & Bailey, 2011). Simple measures may reduce potential barriers and improve participation in lifestyle interventions (Robson & Gray, 2007). When trying to inspire service users in mental health services, it is important to make physical activity as intrinsically motivating as possible by focusing on the positive experiences of the activity itself, as well as helping to develop daily routines for physical exercise (Sörensen, 2005). Common barriers to physical activity have been identified as limited experience of physical activity engagement: the impact of the mental ill health and effects of medication, in addition to discrimination and safety concerns (Johnstone, Nicil, & Donaghy et al., 2009; McDevitt, Snyder, & Miller et al., 2006). Master’s level mental health nurses can implement strategies to make the person feel capable to achieve a healthier lifestyle, as the opposite experience could lead to a feeling of failure and result in avoidance behaviour related to physical activity (Wärdig, Bachrach-Lindström, & Lindström et al., 2015). Support regarding lifestyle changes can focus on strengthening the person’s self-efficacy based on the person’s own experiences, as the meaning of lifestyle changes can be understood as a person’s internal and external endeavours, to make well-considered decisions (Lundström, Hedman Ahlström, & Jormfeldt et al., 2017). Thus, it may be crucial to emphasize a moderate intervention level that facilitates participation and social interactions among group members to perceive oneself as a capable person (Roberts & Bailey, 2011). Nurse-based physical healthcare in mental health services is in its early stages (Happel, Platania-Phung, & Scott, 2014a), and master’s level mental health nurses have an important task to assist service users to understand and verbalize potential physical health risks, and to find out what motivates them to adopt health behaviours (Hultsjö & Syrén, 2013). Studies are showing that further skills and training are required among master’s level mental health nurses to gain a holistic approach including empowering service recipients to engage in realistic, innovative, and pragmatic solutions (Graham et al., 2013; Happell, Platania-Phung, & Scott, 2014b; Happell, Scott, & Platania-Phung et al., 2012). This implies the master’s level mental health nursing competence is essential to support equal health among mental health service users.

### Teamwork and collaboration skills to counteract barriers to a healthy lifestyle among mental health service users

Stigmas from medical staff and social isolation, resulting in a lack of support for engaging in activities to improve physical health, have been described as barriers to physical health care among persons with mental health issues (Kemp, Fisher, & Lawn et al., 2014). Physical activity has shown to enhance mental well-being, physical health and provide social opportunities among mental health service users (Montejo, 2010). Accordingly, physical healthcare needs to be prioritized alongside mental healthcare as a way to improve the long-term outcomes of treatment in mental health services (Montejo, 2010). Programmes to support a healthy lifestyle in mental health services need to be flexible and adaptive to individual needs and, if needed, integrated with assistance from community workers so that the purpose and importance of the programme are explicit and mutually understood (Verhaeghe, Clays, & Vereecken et al., 2013b). The main enabling factors to support participation in physical activity among individuals with severe mental illness have been shown to be the support of the mental health staff and organization and structure of the care organization (Hodgson et al., 2011). Master’s level mental health nurses need to have the skills to communicate lifestyle issues in such a way that it is adjusted to the person’s cognitive ability and the talent to supervise other professional groups to provide an empathic and seriously committed community-based social support (Bergqvist, Karlsson, & Fodemo et al., 2013). Master’s level mental health nurses need to be able to cooperate with health care providers, health agencies and the public in order to support the achievement of a healthy lifestyle, and support the individual with mental illness to enjoy increased social integration (Wärdig, Bachrach-Lindström, & Foldemo et al., 2013),

## Attitudes

Two main attitude aspects needed among master’s level mental health nurses addressed in the literature as prerequisites to support equal health among service users in mental health care are: engagement in person-centred nursing practice to promote overall health and commitment to quality improvement in health promotion in mental health nursing.

### Engagement in person-centred nursing practice to promote overall health

Mental health nurses, as the largest professional group working in mental health care, have a key role in supporting enhanced physical health and well-being of service users (Happell et al., 2014b; Verhaeghe et al., 2013a). Interventions to support a healthy lifestyle among mental health service users, which comprise health education, screening, lifestyle programme delivery and co-ordination of care, have been shown to improve perceived mental functioning, social functioning and self-esteem (Happel et al., 2014a). Nurse-led strategies to improve physical healthcare in mental health services such as lifestyle programmes, screening and linking to general health services could potentially reduce health inequalities (Happell et al., 2014b). Through health-promotion strategies, alongside recovery-focused support aimed at avoiding deteriorating physical health, mental health nurses can significantly counteract the current rate of premature death experienced by people with long-term mental illness (Blythe & White, 2012; Hardy & Thomas, 2012). Nevertheless, role ambiguities among mental health nurses have been revealed, and mental health nurses normally feel that physical care is outside their remit and expertise (Happell et al., 2012). Thus, master’s level mental health nurses should be routinely supported with physical health-care education and training to avoid role ambiguity regarding physical health issues among mental health service users (Blythe & White, 2013; Happell, Platania-Phung, & Scott, 2014c; Happell, Scott, & Hoey et al., 2014d).

### Commitment to quality improvement in health promotion in mental health nursing

Professionals working in psychiatry consider health promotion as an important part of mental health nursing (Happel et al., 2012; Verhaeghe et al., 2011) but clearly defined responsibilities among different professions regarding health promotion in mental health are vague. Studies have shown that people experiencing mental illness often want to learn more about healthy living and be more psychically active (Leutwyler et al., 2014; Verhaeghe et al., 2011). Healthcare organizations can promote provider and patient empowerment by implementing appropriate programmes to support a healthy lifestyle among clients and stimulate staff members to involve clients in treatment planning and ensuring that staff have sufficient time to complete the responsibility of engaging patients (Linhorst, Hamilton, & Young et al., 2002). Master’s level nurses working in mental healthcare settings have an important and crucial role to implement policies and to promote the physical health of people with severe mental illness (Hardy & Thomas, 2012; Usher et al., 2006; Wärdig et al., 2013). Consequently, masters’ level mental health nurses commitment to physical health outcomes of healthcare programmes are essential to reach improvements in health behaviours such as substance use and cardio-metabolic health measures such as cholesterol, blood pressure and blood glucose, among service users in mental health services (Happell, et al., 2014a). Mental health nurses have reported lack of time and personal attitudes as important elements influencing the way health promotion is integrated in the care provided in mental health services (Verhaeghe et al., 2013a). The competence of mental health nurses involves the attitude that all patients require access to health services that do not differentiate or discriminate between people (Shiner, Whitley, & Van Citters et al., 2008). Accordingly, master’s level mental health nurses’ commitment to support equal health among mental health services users includes the attitude to involve a holistic approach, where the service user’s needs for continuity in supportive relationships are being met rather than just referring to the specific symptoms of mental illness (Gunmo & Fatouros Bergman, 2011).

## Conclusions

Responsibilities regarding health-promotion interventions in mental health services remain vague and the stigma associated with mental illness is widespread, even among mental health professionals (Ehrlich et al., 2014; Jormfeldt & Hallén, 2016). Studies show that insufficient priority is currently being given to meet the physical health-care needs of people experiencing mental illness (Happell et al., 2014d; Heald et al., 2010; Laursen & Nodentoft, 2006). Furthermore, the current fragmentation of the health care system entails that individuals with impaired ability to express their needs become at risk of being offered separate treatments for different types of health problems, even though their health issues may have derived from the complexity of the person’s entire life situation (Happell et al., 2012). The service user could be provided treatment for depression by one professional expert and simultaneously be provided with treatment for metabolic syndrome by another, in two different specialized health care settings, of which none has the overall responsibility to consider the service user as a whole person in his or her context. Mental health nurses’ attempts to develop a holistic care by re-integrating services are easily hampered by the fragmentation of general health care and mental health care (Happell et al., 2012). In accordance with the current biomedical healthcare system worldwide, mental health nurses primarily have been found to have the confidence to carry out routine physical health checks but being less confident in health screening and interpreting results or independently promoting physical health among service users (Happel et al., 2014c). Further, mental health nurses far too often still hold a traditional view of being subordinate to other professionals and that physical care is outside their remit and expertise resulting in nurses not taking full responsibility regarding service users’ overall health needs (Happell et al., 2012).

A multidimensional holistic concept of health as a theoretical foundation in mental health nursing provides the opportunity to meet service users’ physical health care needs regardless of other professions’ priorities. Master’s level competencies of mental health nurses’ in terms of KSAs based on a multidimensional holistic concept of health are needed at both individual and organizational levels of administrations, communities and policies regarding mental health services. The specific competencies required among master’s level mental health nurses relates to the ability to acknowledge and bridge nursing theories of positive aspects of holistic health into health-promotion activities through mental health nursing. Master’s level mental health nurses have to be visible as a resource for support and education regarding physical health in mental health care. Master’s level mental health nurses, as a major professional group in mental healthcare, are critical in re-organizing mental health services to better support people with mental illness in their recovery and achievement of equal physical health and well-being.

### Implications regarding master’s level mental health nursing education curricula development in European countries

The discussion of this paper revealed gaps between nursing goals, according to theories of health from a multidimensional holistic perspective and the current praxis as presented in resent studies on the topic of physical health among service users in mental health services. Accordingly, the master’s level mental health nursing education curricula need to focus on knowledge of how nursing theories could be further applied in nursing praxis to change the present situation of the accelerating escalation of physical diseases among mental health service users. The master’s level mental health nursing education curricula need to prepare students by assisting them in learning and developing skills to address therapeutic and motivating skills and skills in teamwork and partnership with other professional caregivers based on frameworks of nursing theories of holistic health. Attitudes of master’s level students need to embrace a significant engagement in person-centred nursing practice to promote overall health, involve a commitment to quality improvement regarding promoting mental health nursing interventions to counteract specific health risks and support equal health among mental health service users.

In conclusion, master’s level mental health nursing education curricula need to integrate knowledge of nursing theories with knowledge of specific health risks among mental health service users. Master’s level mental health nursing education curricula need to be further developed to provide students therapeutic and motivating skills as well as skills in teamwork with other professional caregivers. Students have to be equipped with the attitude of accountability regarding equal health among mental health service users and a profound awareness of the impact of mental health stigma on different levels in our society.
